# Differential Effect of 2 Hormonal Contraceptives on the Relative Telomere Length of Youth With Type 1 Diabetes

**DOI:** 10.1210/jendso/bvae091

**Published:** 2024-05-07

**Authors:** Andrea Castro, M Cecilia Lardone, Franco Giraudo, Patricia López, Eliana Ortiz, Germán Iñiguez, Fernando Cassorla, Ethel Codner

**Affiliations:** Institute of Maternal and Child Research, School of Medicine, University of Chile, Santiago 8360160, Chile; Institute of Maternal and Child Research, School of Medicine, University of Chile, Santiago 8360160, Chile; Institute of Maternal and Child Research, School of Medicine, University of Chile, Santiago 8360160, Chile; Hospital Clínico San Borja Arriarán, Servicio de Salud Metropolitano Central, Santiago 8360160, Chile; Institute of Maternal and Child Research, School of Medicine, University of Chile, Santiago 8360160, Chile; Hospital Clínico San Borja Arriarán, Servicio de Salud Metropolitano Central, Santiago 8360160, Chile; Institute of Maternal and Child Research, School of Medicine, University of Chile, Santiago 8360160, Chile; Institute of Maternal and Child Research, School of Medicine, University of Chile, Santiago 8360160, Chile; Institute of Maternal and Child Research, School of Medicine, University of Chile, Santiago 8360160, Chile; Institute of Maternal and Child Research, School of Medicine, University of Chile, Santiago 8360160, Chile; Centro de Investigación Clínica Aplicada (CICA), School of Medicine, University of Chile, Santiago, Chile

**Keywords:** oral combined contraceptive, subdermal progestin implant, relative telomere length, adolescents and young adults, type 1 diabetes

## Abstract

**Context:**

Adolescents and young women (AYA) with type 1 diabetes (T1D) may require hormonal contraception for an extended period. However, it is unclear what effect hormonal contraception has on telomere length, a marker of the risk for complications.

**Objective:**

To investigate the relative telomere length (RTL) in AYA with T1D (AYA-T1D) and healthy young women (AYA-C) after 18 months of combined oral contraception use (COC) with ethinyl estradiol/desogestrel, or a subdermal etonogestrel implant (IM).

**Methods:**

A nonrandomized prospective study was performed in which 39 AYA-T1D and 40 AYA-C chose the COC or the IM. RTL was measured by monochrome multiplex–quantitative PCR in DNA from peripheral blood mononuclear cells (PBMC). The impact of contraceptives and clinical variables on RTL was assessed using lineal regression analysis.

**Results:**

Longer RTL compared to baseline was observed in AYA-T1D (*P* < .05) and AYA-C (*P*  *<* .01) after using the IM. However, the total of AYA and the AYA-C group treated with COC decreased RTL after 18 months of treatment compared to baseline (*P* < .05). The type of contraceptive used was determinant for the changes in RTL compared to baseline in all subjects and controls (*P ≤ .*006). For AYA-T1D, HbA1c levels were not associated with RTL, but the high-sensitivity C-reactive protein was negatively related with the changes in RTL at 18 months compared to baseline (standardized *R^2^*: 0.230, *P*  *=* .003).

**Conclusion:**

IM was associated with longer RTL in AYA-T1D and AYA-C. In contrast, a shortening of telomere length in PBMC was observed after using COC.

Type 1 diabetes (T1D) is a chronic condition associated with the risk of microvascular and macrovascular complications [1, 2]. Improvements in glycemic levels have been observed in patients with T1D who use modern therapies, leading to better life expectancy and lower prevalence of complications [2]. However, conditions associated with natural aging may occur earlier in persons with T1D [3, 4].

Telomere length attrition is a biomarker of aging [5, 6]. Previous studies have shown an association of shorter telomeres with conditions that display subclinical chronic inflammation, such as type 2 diabetes, insulin resistance, neurodegenerative and kidney diseases, endometriosis, and cardiovascular disorders [5-12]. In persons with T1D, telomere attrition has been associated with a higher risk of morbidity, due to the progression of diabetic complications such as nephropathy, and independently associated with increased mortality [5, 13-15].

On the other hand, women living with T1D face a myriad of reproductive issues during their fertile life [16-18]. Conception in women with T1D should occur with optimal glycemic control [19]. Consequently, contraception is an essential tool for preventing unplanned pregnancies with suboptimal glycemic levels in women with T1D [19-21]. However, there is no information regarding the potential effect of hormonal contraception on chronic subclinical inflammation and telomere length in T1D [22]. To answer this question, we performed a longitudinal, prospective study comparing relative telomere length and subclinical inflammation after the use of a combined oral contraceptive (COC) and a subcutaneous implant (IM) in 39 young women with T1D and a group of 40 matched controls during an 18-month study.

## Methods

### Subjects

Seventy-nine adolescents and young adult women (AYA) aged 15 to 25 years were enrolled. Adolescents and young adults living with T1D (AYA-T1D, n = 39) were recruited from 3 hospitals in Santiago, Chile, and in educational activities sponsored by the Juvenile Diabetes Foundation (JDF) of Chile (www.diabeteschile.cl), an educational nonprofit organization [23]. All the young women treated in the hospitals and JDF were invited by their treating physicians to participate. The 3 hospitals care for middle- and low-income patients living in the neighborhoods assigned to these centers. Similarly, the young women who participated in JDF were treated in the National Public Health System and are from families with low or middle incomes. All the patients who fulfilled the inclusion/exclusion criteria and who signed informed consent could participate. The group invited and included in the trial was representative of Chile based on health insurance, an excellent proxy of socio-economic status in Chile (data not shown).

The young women without diabetes also lived in the same neighborhoods and were looking for contraception in a well-known nonprofit center located in Central Santiago that offers contraception at low cost.

Inclusion criteria were T1D was diagnosed by severe insulinopenic diabetes treated with insulin from the time of diagnosis. All patients were diagnosed at least 1 year before this study and were seeking COC or IM for pregnancy prevention or regulating menstrual cyclicity. The exclusion criteria were as follows: having type 2 or another type of diabetes; being in the honeymoon period, defined as a daily insulin requirement below 0.5 IU/kg/day and glycated hemoglobin (HbA1c) < 53 mmol/mol (< 7%) or > 108 mmol/mol (> 12%), presence of micro/macroangiopathy, T1D duration longer than 20 years, and an HbA1c level greater than 12%.

A group of AYA with normoglycemia was studied as a control group (AYA-C; n = 40) and were recruited at the Chilean Institute of Reproductive Medicine (www.icmer.org) in downtown Santiago. These healthy women were also seeking the provision of contraception and were selected by convenience sampling, too.

AYA-T1D or AYA-C who had used oral hormonal contraceptives in the last 2 months before entering the study, used injectable contraception in the previous 6 months, or who were breastfeeding, pregnant, or had given birth less than 1 year ago, were excluded. Concomitant chronic conditions, a familiar history of thromboembolism, as well as the use of medications were also exclusion criteria. Mild compensated hypothyroidism or asthma were not considered as exclusion criteria.

Signed informed consent of the parents and young adults and informed assent from adolescents were obtained from all study participants, which was approved by the Ethics Committee on Research with Human Beings of the University of Chile, following the guidelines of the Helsinki Declaration.

### Study Design

A prospective comparison of 2 contraceptive methods was carried out. The study was designed following the CHOICE study [24]. Both groups of women chose between a progestin IM (etonogestrel 68 μg, Implanon®) or a COC with 150 μg of desogestrel (DSG) and 30 μg of ethinyl estradiol (EE). DSG is metabolized to the active metabolite etonogestrel (ETO); therefore, the progestin used was similar in both methods. The subjects were studied between September 2017 and March 2021. Metabolic aspects related to diabetes management have recently been published [25]. The trial was registered in the ISRCTN registry with the code ISRCTN29256651.

Based on body mass index (BMI) levels, women were classified as having normal or excessive weight. For adolescents younger than 18 years, a BMI greater than the 85th percentile was defined as excessive weight, and a BMI greater than 25 kg/m^2^ was defined as excessive weight for women older than 18 years.

The measurements of high sensitive C-reactive protein (hs-CRP), arterial pressures (systolic, diastolic and mean), lipids (triglycerides, low-density lipoprotein cholesterol [LDL-C], high-density lipoprotein cholesterol [HDL-C] and total cholesterol), as well as the calculation of SD scores of weight (z-weight), height (z-height), BMI (z-BMI), the proportion of total body fat, and daily and total insulin dose were performed as previously described [25].

### Molecular Study

The relative telomere length (RTL) was determined at baseline before the initiation of the contraceptive method and after 12 and 18 months of contraceptive use. The deltas (Δ) of the RTL at the end of 12 and 18 months of contraceptive use were referred to as RTL Δ18-0 and RTL Δ12-0.

We studied the RTL on peripheral blood mononuclear cells (PBMC) DNA using the monochrome multiplex–quantitative chain reaction (MM-qPCR). PBMC were isolated on a gradient of HISTOPAQUE®-1.077 (Sigma-Aldrich) and immediately used for genomic DNA extraction (Puregene® Core kit A, Qiagen). Subsequently, extracted DNA was aliquoted for storage at −80 °C until quantification of telomere length relative was performed. We followed the MM-qPCR method described by Cawthon et al [26] with some modifications introduced by Jiao et al [27]. Briefly, PCR reactions were carried out in a final volume of 20 µL containing 10 µL of QuantiFast SYBR Green PCR Master Mix (QuantiFast SYBR Green PCR, Qiagen), 20 ng of DNA diluted once at 5 ng/μL immediately before use, and the primer pairs telg/telc (900 nM each) and hbgu/hbgc (500 nM each) for the respective amplification of telomeres and β globin amplification as previously described by Cawthon et al [26]. The optimized thermal cycling profile included the following stages [27]: (i) 15 minutes at 95 °C; (ii) 2 cycles of 15 seconds at 94 °C, and 60 seconds at 49 °C; (iii) 4 cycles of 15 seconds at 94 °C, 30 seconds at 62 °C; (iv) 20 cycles 15 seconds at 84 °C, 30 seconds at 62 °C and 15 seconds at 73 °C with signal acquisition (providing Ct values for the amplification of tel); and (v) 27 cycles of 15 seconds at 94 °C, 10 seconds at 84 °C and 15 seconds at 87 °C with signal acquisition (providing Ct values for the amplification of β-globin). All reactions were performed in triplicate, and replicates ≥ 0.5 Ct beyond the mean were excluded. Reaction efficiency was calculated using the slope of standard curves generated by amplifying 5 serial dilutions of reference DNA from 152 to 4.75 ng, achieving an efficiency of 97% ± 2.9% and 103% ± 4.5 for telomere and β globin amplification, respectively. The RTL for each DNA sample was calculated using the 2^−ΔΔCt^ method, where a 27-year-old healthy volunteer DNA was used as the reference sample. Inter-assay variability was assessed by amplifying 3 different standard samples in every experiment (n = 15), and the mean coefficient of variation was 15.9% ± 1.6%.

The validation method used for the determination of RTL was similar to the validation described by Cawthon et al [26], which showed no overlap in the telomere repeats and beta-globin amplification signals (Ct threshold). Moreover, we used a DNA sample from an 80-year-old woman as a case with short RTL control and observed a threshold Ct for telomere 3 to 4 cycles earlier than the threshold Ct for the beta-globin gene. To compare the RTL obtained by MM-qPCR method with the data obtained by another methodology, we measured the RTL by flow cytometry and fluorescence in situ hybridization (Flow-FISH) using a fluorescein-conjugated PNA probe (K5327, Telomere PNA Kit/FITC, DAKO) in 59 vital frozen basal samples of PBMC (31 COC and 28 IM users similarly distributed between T1D and controls and randomly selected). A significant correlation was observed between the 2 techniques (Spearman correlation coefficients of 0.532, 0.570, and 0.423; *P*  *=* .00002, *P*  *=* .001, and *P*  *=* .025, respectively, for all subjects, T1D, and controls).

### Statistical Analysis

Based on the study of changes in telomere length in adolescents using COC [22] with an error α of .05, a statistical power of 80% and a SD of 0.4, a minimal sample size of 12 AYA per group was calculated.

The normal distribution of the variables was assessed with Shapiro-Wilk. RTL, RTL Δ12-0, and RTL Δ18-0 had normal distribution (*P* > .05) and were evaluated with parametric statistics. Student *t* test was used for the analysis of continuous variables, and the Fisher exact test was used for the assessment of differences in proportions. Paired Student *t* test was used to evaluate differences in RTL at 12 and 18 months compared to baseline. Comparison of RTL Δ12-0 and RTL Δ18-0 in the IM vs COC group was assessed with a Student *t* test.

Linear regression was used to examine possible associations of RTL Δ 12-0 and Δ RTL 18-0 with the type of contraceptive and/or the change of the mean determinations of the high-sensitivity C-reactive protein (hs-CRP Δ), z-BMI (z-BMI Δ), and the average of mean arterial pressures (MAP_mean) at basal and 12 and 18 months of treatment in all subjects, which were stratified by the presence of diabetes.

## Results

The clinical characteristics of the subjects before starting their corresponding contraceptive treatment are shown in Table 1. The AYA-T1D and AYA-C groups had similar age, BMI, body fat, and age at menarche. The clinical features of the subjects at 12 and 18 months after starting treatment are shown in Supplementary Table S1 [28]. As expected, higher HbA1c, systolic and diastolic blood pressure, MAP, and hs-CRP were observed in AYA-T1D than in AYA-C. In addition, the AYA-T1D treated with COC (T1D-COC) at baseline had higher levels of total cholesterol, LDL-C, and HDL-C compared with the AYA-C COC treated (C-COC) (*P*  *<* .005), but in the follow-up, this difference was only observed for LDL-C at 18 months. The hs-CRP levels were higher in T1D-COC and C-COC groups after 12 months of treatment (*P* < .01, Wilcoxon test) [28].

**Table 1.** Baseline clinical characteristics and laboratory measurements by contraceptive treatment group

Characteristics	Implant			COC		
	T1D	Control	*P value* ^#^	T1D	Control	P value*
	(n = 19)	(n = 19)		(n = 20)	(n = 21)	
Age, y	19.3 ± 3.0	19.0 ± 3.0	.770	20.6 ± 3.3	20.3 ± 2.9	.827
Weight, z score	0.35 ± 1.1	−0.04 ± 0.9	.105	0.51 ±0 .6	0.21 ± 0.8	.172
Height, z score	−0.85 ± 0.9	−0.74 ±0 .8	.942	−0.53 ± 0.6	−0.35 ± 0.6	.329
BMI, kg/m^2^	24.6 ± 3.7	22.7 ± 3.5	.118	25.0 ± 3.0	23.2 ± 3.1	.078
BMI, z score	0.87 ± 0.9	0.35 ± 1.1	.108	0.97 ± 0.7	0.49 ± 0.9	.067
Body fat, %	29.4 ± 7.8	25.5 ± 7.0	.116	29.6 ± 5.1	27.0 ± 6.6	.168
HbA1c, %	7.6 ± 1.1	5.2 ± 0.1	<.001	7.8 ± 0.9	5.3 ± 0.21	<.001
Duration of diabetes, y	8.4 ± 3.6			9.5 ± 3.6		
Total daily insulin dose, UI/kg	0.97 ± 0.3			0.98 ± 0.4		
Age at menarche, y	12.1 ± 1.6	12.0 ± 1.8	.940	11.7 ± 1.1	12.3 ± 1.5	.148
SBP, mmHg	113 ± 12	105 ± 10	.039	115 ± 10	108 ± 9	.030
DBP, mmHg	74 ± 8	63 ± 7	<.001	75 ± 9	63 ± 7	<.001
MAP, mmHg	87 ± 6	77 ± 7	<.001	88 ± 9	78 ± 7	<.001
Total cholesterol, mg/dL	157 ± 20	150 ± 25	.325	164 ± 37	139 ± 30	.020
LDL-C, mg/dL	85 ± 19	79 ± 24	.403	89 ± 28	73 ± 27	.042
HDL-C, mg/dL	54 ± 12	51 ± 8	.328	59 ± 18	49 ± 10	.034
Triglycerides, mg/dL	92 ± 48	101 ± 41	.470	79 ± 45	86 ± 37	.442
hs-CRP, ng/mL	4.7 ± 4.0	0.87 ± 0.6	<.001	3.1 ± 2.4	1.8 ± 2.2	.023
Excessive weight, n (%)	9 (47)	4 (21)	.170	11 (55)	6 (29)	.118

Data represent mean ± SD. Comparison between T1D and Control group treated with IM (#) or COC (*), calculated by Mann-Whitney or Student *t* test for continuous variables or Fisher exact test for proportion of categorical variable *Excessive weight* (overweight and obesity). Abbreviations: BMI, body mass index; COC, combined oral contraceptive; DBP, diastolic blood pressure; HbA1c, glycated hemoglobin; HDL-C, high-density lipoprotein cholesterol; hs-CRP, high-sensitivity C-reactive protein; LDL-C, low-density lipoprotein cholesterol; MAP, mean arterial pressure; SBP, systolic blood pressure; T1D, type 1 diabetes.

**Figure 1.**
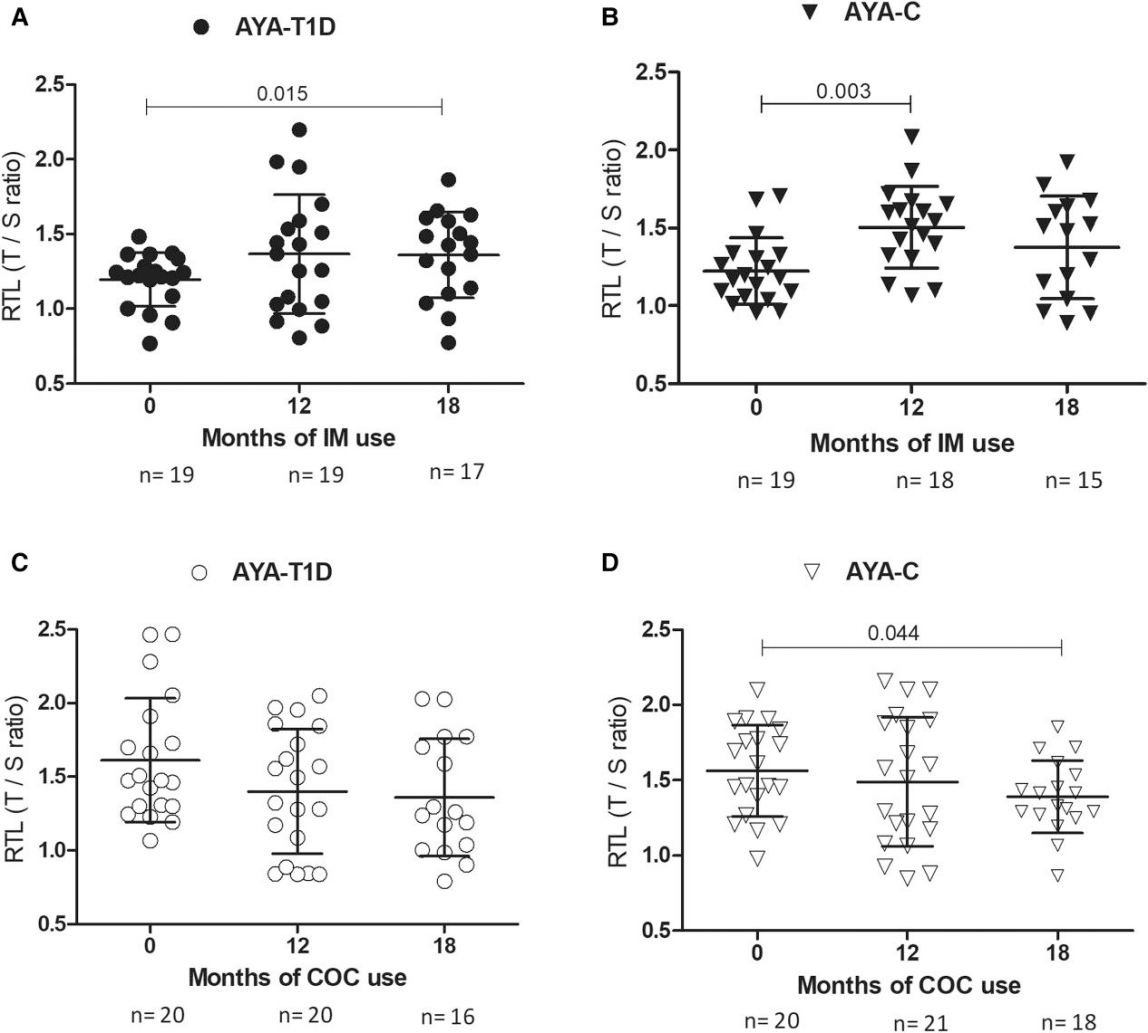
Relative telomere length of peripheral blood mononuclear cells in contraceptive users of implant (IM) or combined oral contraceptive (COC) at baseline and after 12 and 18 months of starting treatment. Numbers above the line marks represent *P* values showing significant differences calculated by Student *t* test for paired samples. T1D and Controls are shown in circles and triangles respectively. IM users are shown in filled symbols and COC in empty symbols. A: T1D-IM. B: C-IM. C: T1D-COC. D: C-COC.

**Figure 2.**
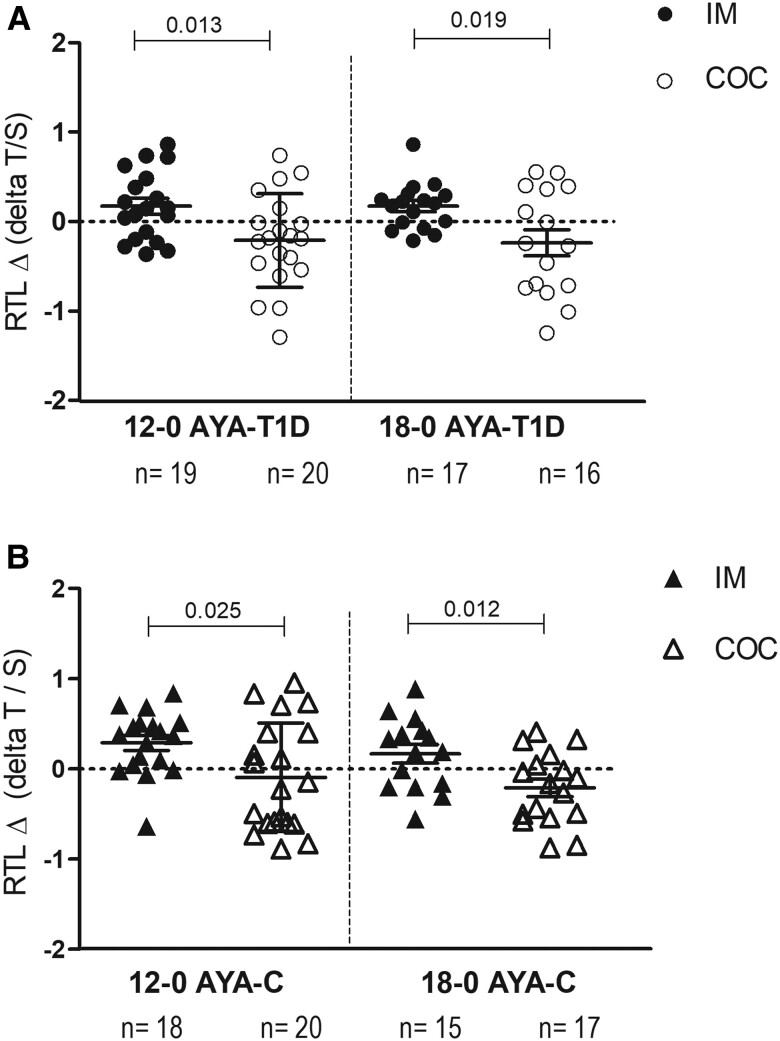
Change in telomere length of peripheral blood mononuclear cells in IM vs COC users. Numbers above the line marks are significant *P* values for comparison between IM (filled) and COC (empty) at 12 or 18 months of treatment, *t* test for independent samples.

The relative telomere length (RTL) is shown in Fig. 1. Longer RTL was observed in T1D and C groups after using the IM, but not in the COC groups. In the T1D-IM group, the RTL was longer compared to baseline after using the IM for 18 months (Fig. 1A, *P* < .05); in the C-IM group, a longer RTL was observed after 12 months (Fig. 1B, *P* < .01). If we consider the total of AYA (T1D and C together) treated with IM, a significantly higher increase of RTL at 12 and 18 months of IM use was observed (*P*  *<* .001 and *P*  *=* .006, respectively). On the other hand, the C-COC group had a decrease in the RTL after 18 months of treatment compared to baseline (*P*  *=* .044; Fig. 2D), and the T1D-COC group had a similar RTL throughout the study (Fig. 2C). When the total of AYA treated with COC was analyzed together, a larger decrease in the RTL was observed (*P*  *=* .014).

RTL Δ12-0 and RTL Δ18-0 are shown in Fig. 2. RTL Δ12-0 and RTL Δ18-0 were significantly different in AYA-T1D using IM compared to the AYA-T1D group using COC (*P*  *=* .013 and *P*  *=* .019, respectively, Fig. 2A). AYA-T1D who used the IM had a positive RTL Δ12-0 and RTL Δ18-0, whereas T1D who used the COC had a negative RTL Δ12-0 and RTL Δ18-0. Similar differences in RTL Δ12-0 and RTL Δ18-0 were observed in the control group who used the IM compared to the control group who used COC (Fig. 2B).

The regression analysis is shown in Table 2 and Table 3. In agreement with the opposite change in RTL observed with the COC and IM treatment (Fig. 2), the type of contraceptive method was a determinant factor in the regression models to predict the RTL Δ12-0 (Table 2), and RTL Δ18-0 (Table 3). The association between the contraceptive and RTL Δ12-0 was observed in the total group of subjects (F: 12.2, *P*  *<* .001), in T1D (F: 6.8, *P*  *=* .013) and in controls (F: 5.6, *P*  *=* .025). For the RTL Δ18-0, the type of contraceptive method was the main determining factor in all subjects and controls, respectively (*P ≤* .004). However, in the T1D group, the change in hs-CRP was the only determining factor predictive for RTL Δ18-0, and this was negatively associated with RTL (F: 10.6, *P*  *=* .003). The remaining variables, such as the presence of T1D, the mean of HbA1c, and the mean of MAP, were not determinant factors of the RTL Δ12-0 and RTL Δ18-0, and therefore, were excluded from the regression models.

**Table 2.** Linear regression models for the change in RTL after 12 months of contraceptive

Predicted variable	Nonstandardized		Standardized	*R^2^*	Sig.	95% CI for B	
	B	Standard Error	Beta			Lower limit	Upper limit
**Total subjects**							
(Constant)	**.228**	**.078**			**.005**	**.072**	**.384**
IM = 0, COC = 1	−**.38**	**.109**	−**.374**	**0.129**	**<.001**	−**.597**	−**.163**
Control = 0, T1D = 1	.01	.142	.009		.946	−.273	.292
hs-CRP_Δ12-0	.013	.012	.122		.294	−.011	.036
z-BMI_Δ12-0	−.066	.113	−.063		.561	−.292	.16
MAP_mean12	−.017	.01	−.246		.094	−.038	.003
**T1D**							
(Constant)	**.172**	**.105**			**.111**	−**.042**	**.386**
IM = 0, COC = 1	−**.383**	**.147**	−**.393**	**0.132**	**.013**	−**.681**	−**.085**
hs-CRP_Δ12-0	.007	.013	.09		.589	−.019	.033
z-BMI _Δ12-0	−.266	.202	−.206		.197	−.676	.145
HbA1c_mean12	.006	.072	.013		.931	−.139	.152
MAP_mean12	−.012	.013	−.147		.38	−.039	.015
**Controls**							
(Constant)	**.287**	**.118**			**.02**	**.048**	**.526**
IM = 0, COC = 1	−**.381**	**.162**	−**.364**	**0.108**	**.025**	−**.71**	−**.051**
hs-CRP_Δ18-0	.03	.035	.14		.399	−.042	.102
z-BMI_Δ12-0	−.002	.15	−.002		.99	−.307	.303
MAP_mean12	−.022	.017	−.211		.192	−.056	.012

The results for the significant models obtained in the linear regression analysis are shown in bold. Abbreviations: COC, combined oral contraceptive; IM, etonogestrel implant; hs-CRP, high-sensitivity C-reactive protein; MAP, mean arterial pressure; T1D, type 1 diabetes; z-BMI, z score of body mass index.

**Table 3.** Linear regression models for the change in RTL after 18 months of contraceptive

Predicted variable	Nonstandardized		Standardized	Standardized	*P* value	95% CI for B	
	B	Standard Error	Beta	*R^2^*		Lower limit	Upper limit
**Total subjects**							
(Constant)	**.150**	**.072**			**.04**	**.007**	**.294**
IM = 0, COC = 1	**−.31**	**.105**	**−.339**	**0.307**	**.004**	**−.52**	**−.1**
hs-CRP_ Δ18-0	−**.022**	**.008**	−**.306**		**.01**	−**.038**	−**.006**
z-BMI_Δ18-0	**.225**	**.095**	**.248**		**.021**	**.034**	**.415**
Control = 0, T1D = 1	.083	.126	.09		.516	−.17	.336
MAP_mean18	−.003	.009	−.048		.739	−.022	.015
**T1D**							
(Constant)	**.101**	**.085**			**.244**	−**.072**	**.273**
hs-CRP_Δ18-0	−**.031**	**.01**	−**.504**	**0.230**	**.003**	−**.05**	−**.012**
IM = 0, COC = 1	−.274	.157	−.283		.093	−.596	.049
HbA1c_mean18	−.04	.068	−.092		.557	−.18	.099
z-BMI_Δ18-0	.176	.15	.18		.252	−.132	.484
MAP_mean18	.001	.013	.011		.947	−.026	.027
**Controls**							
(Constant)	**.167**	**.100**			**.107**	−**.038**	**.371**
IM = 0, COC = 1	−**.417**	**.139**	**−.486**	**0.210**	**.006**	−**.703**	−**.132**
hs-CRP_Δ18-0	−.008	.021	−.078		.708	−.051	.035
z-BMI_Δ18-0	.29	.138	.343		.045	.007	.573
MAP_mean18	−.013	.015	−.139		.39	−.043	.017

The results for the significant model obtained in the linear regression analysis are shown in bold. Abbreviations: COC, combined oral contraceptive; IM, etonogestrel implant; hs-CRP, high-sensitivity C-reactive protein; MAP, mean arterial pressure; T1D, type 1 diabetes; z-BMI, z score of body mass index.

According to the regression models described in Table 2, significant correlations between the calculated and predict value of RTL Δ12-0 were observed in total subjects (0.374, *P* < .001), T1D (R: 0.393, *P* = .022), and AYA-C (R: 0.364, *P*  *=* .025). Regarding RTL Δ18 (Table 3), higher correlations were observed in total subjects (0.584, *P*  *<* .001), in T1D (0.504, *P*  *=* .003), as well as in controls (0.439, *P*  *=* .012).

## Discussion

This study investigated the change in RTL associated with the use of 2 types of hormonal contraception in AYA with T1D, as well as a control group without diabetes. We studied 79 AYA receiving either COC or IM for 18 months. The type of contraceptive was the main factor that affected the changes in RTL in both groups of AYA women, with COC associated with a shorter RTL, whereas the IM group had a longer RTL. The widely used MM-qPCR methodology was used to assess the RTL in mononuclear blood cells after validation with the flow-FISH methodology.

A relevant finding of this study is the increase in RTL observed with the use of the ETO implant. This contraceptive method lacks estrogen and releases ETO to the systemic circulation, bypassing *first-step* hepatic metabolism and leading to persistently elevated serum levels of ETO [9, 13, 29, 30]. ETO reaches a steady state of 200 pg/mL after 4 months following insertion of the implant and remains sufficient to prevent ovulation for 3 years [30, 31]. The ETO implant acts as a contraceptive by inhibiting ovulation, but the ovarian follicular activity and estradiol levels are not suppressed (24, 26). Previous studies have demonstrated normal estradiol levels after 6 months of use [30, 31].

Recent research in animal models and human beings suggests that progesterone administration may have beneficial effects. A study in diabetic rats showed that progesterone has antioxidant and anti-inflammatory protective effects and is associated with reduced neuronal damage [32]. Studies in patients with autoimmune and inflammatory diseases show that progesterone administration has immunomodulatory actions and regulates the balance between pro- and anti-inflammatory cytokines [33].

The fact that the IM use was associated with lengthening of the RTL may be explained by the positive metabolic effects of ETO. Hernández-Juarez et al showed that the ETO implant in AYA (18-35 years of age) was associated with positive metabolic effects on glucose, hs-CRP, and lipid levels, which were not observed with the use for 4 months of a contraceptive skin patch containing EE and norelgestromine [34]. Similarly, we observed that hs-CRP levels were negatively associated with RTL Δ 18-0. Recently, we showed that serum hs-CRP levels were higher after 6 months of COC use compared with IM [25].

Because the progestin we used was similar for both contraceptives, our data suggest that the oral administration of EE is the key factor for the shortening of the RTL. However, several studies have reported higher telomerase activity and longer telomeres when exposed to higher estrogen levels in vivo [35]; in vitro, studies have shown that estrogen may have direct and indirect stimulatory effects on the expression of telomerase in PBMC, ovarian human epithelial, benign and cancer prostatic cells, and the MCF7 breast cancer cell line [35-41]. Therefore, we hypothesize that the shortening of telomeres in COC users was associated with harmful direct inflammatory hepatic effects of the EE, which oppose a probable greater telomerase-stimulating action, given its known reduced hepatic metabolism and higher potency compared to estradiol [42-44]. The use of COC-containing EE has been shown to have detrimental effects on body composition, inflammation, and hepatic synthesis of several coagulation factors [43, 45].

Clinically, a decrease in RTL in persons living with T1D is a nondesirable outcome that may be a point to consider in the selection of the type of contraception in AYA-T1D [46]. Previously, the Wisconsin Epidemiologic study showed that COC does not increase the rate of retinopathy [45] or cardiovascular mortality [47]. However, studies evaluating the effect of currently available COC therapy on the risk of developing micro- and macrovascular complications in AYA with T1D currently with modern diabetes therapy are not available [48].

Several strengths of our study should be highlighted. The investigation had a prospective design, using 2 commonly used hormonal contraceptive methods, an ETO subdermal implant and a COC with EE and DSG. The recruitment of AYA with a confirmed diagnosis of T1D and a group of healthy controls in a prospectively designed trial is an important aspect of this study. Previous contraception studies have evaluated older women, including women with T1D and type 2 diabetes with a wide age range, and none have studied exclusively youth with T1D. One of the limitations of our study is that some AYA were lost to follow-up due to the SARS-COV2 pandemic.

In conclusion, our data show for the first time that the prospective use of a COC for 18 months is associated with the shortening of telomere length in mononuclear blood cells of AYA, which may be particularly detrimental for patients with T1D. EE may mediate this adverse effect in the COC. In contrast, the use of a progestin subdermal implant was associated with an increase in the RTL after 12 or 18 months in both AYA with T1D, and AYA with normal glucose levels. These data suggest that the subdermal progestin implant is a good contraceptive option for AYA with or without T1D.

## Data Availability

The data generated and analyzed during this study may be available to the corresponding author upon reasonable request.

## Clinical Trial Information

Clinical trial registration number: ISRCTN29256651

## Abbreviations

AYA: adolescents and young women
BMI: body mass index
COC: combined oral contraceptive
DSG: desogestrel
EE: ethinyl estradiol
ETO: etonogestrel
HbA1c: glycated hemoglobin
HDL-C: high-density lipoprotein cholesterol
IM: etonogestrel implant
hs-CRP: high-sensitivity C-reactive protein
JDF: Juvenile Diabetes Foundation
LDL-C: low-density lipoprotein cholesterol
MAP: mean arterial pressure
MM-qPCR: monochrome multiplex–quantitative chain reaction
PBMC: peripheral blood mononuclear cells
RTL: relative telomere length
T1D: type 1 diabetes
z-BMI: z score of BMI
